# A Soybean C2H2-Type Zinc Finger Gene *GmZF1* Enhanced Cold Tolerance in Transgenic *Arabidopsis*

**DOI:** 10.1371/journal.pone.0109399

**Published:** 2014-10-06

**Authors:** Guo-Hong Yu, Lin-Lin Jiang, Xue-Feng Ma, Zhao-Shi Xu, Meng-Meng Liu, Shu-Guang Shan, Xian-Guo Cheng

**Affiliations:** 1 Key Lab. of Plant Nutrition and Fertilizer, Ministry of Agriculture, Institute of Agricultural Resources and Regional Planning, Chinese Academy of Agricultural Sciences, Beijing, China; 2 Institute of Agro-Products Processing Science and Technology, Chinese Academy of Agricultural Sciences, Beijing, China; 3 Institute of Crop Science, Chinese Academy of Agricultural Sciences (CAAS)/National Key Facility for Crop Gene Resources and Genetic Improvement, Key Laboratory of Biology and Genetic Improvement of Triticeae Crops, Ministry of Agriculture, Beijing, China; Institute of Genetics and Developmental Biology, Chinese Academy of Sciences, China

## Abstract

Zinc finger proteins were involved in response to different environmental stresses in plant species. A typical Cys2/His2-type (C2H2-type) zinc finger gene *GmZF1* from soybean was isolated and was composed of 172 amino acids containing two conserved C2H2-type zinc finger domains. Phylogenetic analysis showed that GmZF1 was clustered on the same branch with six C2H2-type ZFPs from dicotyledonous plants excepting for GsZFP1, and distinguished those from monocotyledon species. The GmZF1 protein was localized at the nucleus, and has specific binding activity with EP1S core sequence, and nucleotide mutation in the core sequence of *EPSPS* promoter changed the binding ability between GmZF1 protein and core DNA element, implying that two amino acid residues, G and C boxed in core sequence TGACAGTGTCA possibly play positive regulation role in recognizing DNA-binding sites in GmZF1 proteins. High accumulation of *GmZF1* mRNA induced by exogenous ABA suggested that *GmZF1* was involved in an ABA-dependent signal transduction pathway. Over-expression of *GmZF1* significantly improved the contents of proline and soluble sugar and decreased the MDA contents in the transgenic lines exposed to cold stress, indicating that transgenic *Arabidopsis* carrying *GmZF1* gene have adaptive mechanisms to cold stress. Over-expression of *GmZF1* also increased the expression of cold-regulated *cor6.6* gene by probably recognizing protein-DNA binding sites, suggesting that *GmZF1* from soybean could enhance the tolerance of *Arabidopsis* to cold stress by regulating expression of cold-regulation gene in the transgenic *Arabidopsis*.

## Introduction

Plants are usually exposed to various environmental stress factors affecting plant growth and crop productivity, such as drought, high salt and low temperature. As an adaptive response, the plants have developed a resistance mechanism to abiotic stresses to achieve an optimal adaptation [1]. It was reported that a number of transcription factor gene from different plants was induced or repressed during the responses and acclimations [2]. For example, some typical transcription factors, AP2/ERF, bZIP, NAC, MYB, MYC, WRKY and zinc finger proteins have been well characterized, and confirmed to be involved in stress response via transcriptional regulation modulation [3], [4], [5], and these transcription factors usually have specific structure and/or conserved domains functioning in the adaptation to abiotic stresses [6], [7].

The first zinc finger transcription factor IIIA (TFIIIA) was recognized as a repeated zinc-binding motif in *Xenopus*, and is a classical Cys2/His2-type (C2H2-type) zinc finger gene [6]. The C2H2-type zinc finger has been well characterized in the eukaryotic transcription factors having specific CX_2-4_CX_3_FX_5_LX_2_HX_3-5_H structure (X: any amino acid; number: amino acid amounts), and this conserved motif contains two pairs of specific Cys and His residues which form a tetrahedral structure with a zinc ion [7]. In plants, the C2H2-type zinc finger has a highly conserved QALGGH motif in a putative DNA-contacting surface, which is unique in zinc-finger proteins from plants [8], [9]. *In* *vitro* analysis revealed that the conserved QALGGH motif in plants plays a critical role in DNA binding activity, and each amino acid residue in the motif seems to be essential for maintaining effective binding between DNA and C2H2-type zinc finger proteins [10], [11], and these specific domains conferred that zinc-finger proteins play important regulatory role by recognizing the target sequences and regulating expression of target gene in a plant-specific manner [1], [8], [12].

As a first C2H2-type zinc finger protein in plants, ZPT2-1 was found in petunia (*Petunia hybrid*), and identified to interact with the specific DNA sequences in the promoter region of 5-enolpyruvylshikimate-3-phosphate synthase (EPSPS) [1], [13]. Thereafter, several TFIIIA-type zinc finger proteins have been consecutively reported [1], [8], and these C2H2-type zinc finger genes paly important regulation roles in responses to various abiotic stresses [8], [9], [14]. In petunia, the TFIIIA-type zinc finger genes, *ZPT2-2* and *ZPT2-3*, were regulated by cold and/or drought, and over-expression of *ZPT2-3* gene in the transgenic petunia increased the tolerance of plants to drought stress [15], [16]. *STZ* is one of the *ZPT2*-related genes in *Arabidopsis*, and was isolated by complementation of the salt-sensitive phenotype with a yeast calcineurin mutant [17], and the expression of *STZ* gene has been identified to be responsive to drought, salt, cold and abscisic acid (ABA), and that constitutive expression of *STZ* resulted in a suppression in growth, and accompanied an enhancement of plants adaptation to drought and osmotic stresses [18], [19], [20].

To date, few zinc finger proteins from cultivar soybean (*Glycine max*) have been isolated and characterized. *SCOF-1,* a typical C2H2-type zinc finger gene, was isolated from soybean and was confirmed to have a positive role in regulating the expression of cold-regulated gene and enhancing cold tolerance in transgenic plants [21]. *GsZFP1*, a zinc finger transcription factor lacking typical QALGGH motif was isolated from wild soybean (*Glycine soja* L. G07256), and transformed into *Arabidopsis*, and confirmed to play a crucial role in withstanding cold and drought stresses [22], indicating that the presence of QALGGH motifs in the C2H2 zinc finger *GsZFP1* gene from wild soybean seems to be not a crucial element in functioning during adaptation to abitotic stress. In our study, a novel C2H2-type zinc finger gene, *GmZF1* was isolated from cultivar soybean, and identified to have two typical conserved QALGGH motifs, and is significantly different from *GsZFP1* gene in wild soybean [22]. To our knowledge, the understanding on the function of zinc finger C2H2 from cultivar soybean in responses to cold stress is still limited. Therefore, based on the structure analysis and *in* *vitro* identification of *GmZF1* gene, we transformed the *GmZF1* gene into *Arabidopsis* plants and identified three homozygous lines which were subsequently used to observe the function effects of *GmZF1* gene in responses to cold stress and exogenous ABA. Our data showed that *GmZF1* from cultivar soybean was induced by ABA, and over-expression of *GmZF1* gene significantly enhanced the tolerance of the transgenic *Arabidopsis* to low temperature stress.

## Results

### Characteristics of the *GmZF1* gene

**Figure 1:**
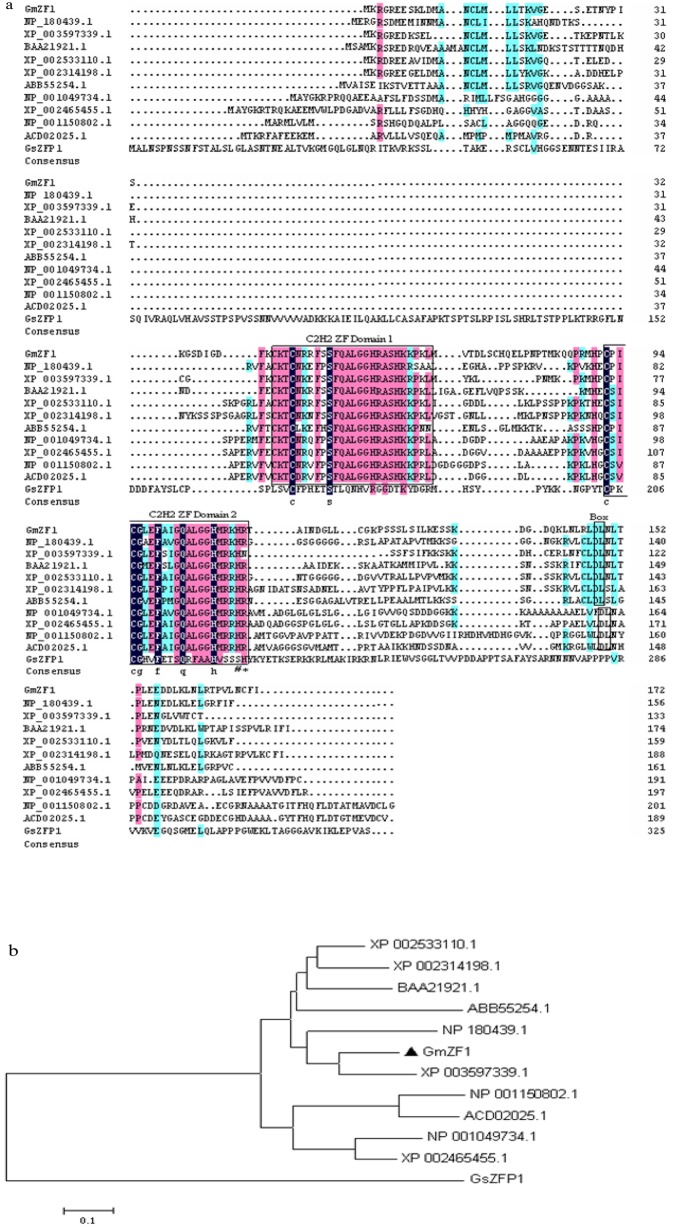
Sequence characteristic of GmZF1 deduced amino acids. a: Multiple alignment of GmZF1 amino acid sequences with XP_003597339.1 (*Medicago truncatula*), BAA21921.1 (*Petunia hybrid*)), NP_180439.1 (*Arabidopsis*) and NP_001049734.1 (*Oryza sativa*). The two zinc finger domains and the conserved DLN amino residues are boxed. b: Phylogenetic analysis of GmZF1 and related proteins. A mid-point rooted neighbor-joining phylogeny was constructed by 12 amino acid sequences from diverse organisms. Excepting for GmZF1, the other C2H2 type zinc finger proteins were demonstrated by XP_002533110.1 (*Ricinus communis*), XP_003597339.1 (*M. truncatula*), BAA21921.1 (*Petunia*), XP_002314198.1 (*Populus*), NP_180439.1 (*Arabidopsis*), ABB55254.1 (*Brassica*), NP_001049734.1 (*Oryza sativa*), XP_002465455.*1* (*Broomcorn*), NP_001150802.1 (*Zea mays*), *ACD0202*5.1 (*Triticum aestivum*) and GsZFP1 (*Glycine sojia*. L).

The *GmZF1* cDNA is composed of 765-bp nucleotides, and eocodes a predicted protein of 172 amino acids with a calculated molecular mass of 19.2 kDa, and flanked by a 97 bp fragment at 5′ end and a 149 bp fragment at 3′ end at two untranslating regions, respectively (accession number DQ055134). Electrophoresis analysis showed that PCR product from the genomic DNA of soybean seedlings has the same size as that from the cDNA length generated by RT-PCR using total RNA as remplate, and sequencing also showed that the generated *GmZF1* clones from gDNA and cDNA have identical sequence, indicating that *GmZF1* gene has no intron in the genomic DNA. A homology search against the GenBank database showed that GmZF1 is a homolog of C2H2-type zinc finger proteins (ZFPs), and has two typical C2/H2 type zinc finger domains (CX_2_CX_3_FX_3_QALGGHX_3_H) (Fig. 1a). GmZF1 has 57%, 48%, 47% and 46% homology with the C2H2-type ZFPs from *Medicago truncatula*, Petunia, *Arabidopsis* and rice, respectively, and no highly homology was observed between these genes from different plants, suggesting that *GmZF1* belongs to a novel subfamily member of C2H2 zinc finger. Like C2H2 zinc finger genes from four kinds of plants mentioned above, the GmZF1 protein also has plant-specific QALGGH motifs as well as a conserved motif L-box with more Leu residues, and contains a short hydrophobic region with a highly conserved DLN box near the C-terminus of GmZF1 (Fig. 1a), which may function as a transcription repression domain [16], [18]. It is noteworthy that GmZF1 have two conserved domains with QALGGH motifs, and is significantly different from *GsZFP1* isolated from wild soybean, the later only has one C2H2 domain lacking QALGGH motif, and the number of amino acid residues between two His (H) residues is composed of four amino acids, which is different from GmZF1 having three amino acids between two His amino acids (as indicated by # and * respectively in Fig. 1a), and no DLN box was found at the C-terminus of GsZFP1 [22].

For profiling the differences in genetic characteristics between C2H2 family members from different plants, a systematic phylogenetic analysis was carried out. The results showed that GmZF1 was clustered on the same branch with six C2H2-type ZFPs from dicotyledonous plants, and distinguished them from monocotyledon species (Fig. 1b), indicating that dicotyledonous plants and monocotyledon plants have significant difference in genetic characteristics of C2H2-type genes, and probably play different regulation roles in plant responses to abiotic stresses. What's worthy reminding is that *GmZF1* from cultivar soybean and *GsZFP1* from wild soybean respectively belong to two different subfamily members in C2H2 zinc finger super family.

### Analysis for SDS-PAGE and DNA-binding activity of GmZF1

**Figure 2:**
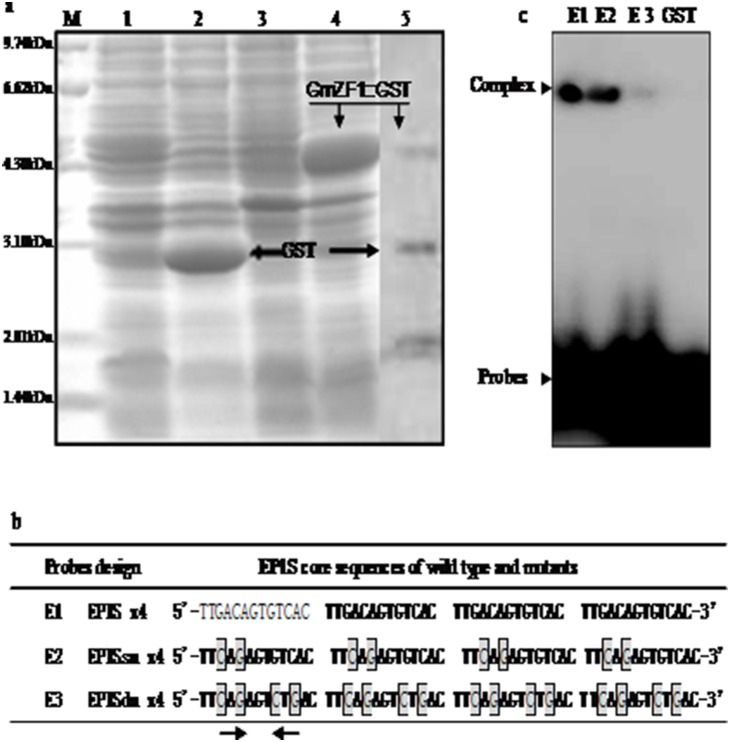
SDS PAGE induction expression and gel shift assay for DNA-binding activities *in* *vitro*. a: Induction expression and purification of target proteins, GmZF1::GST fusion protein and GST protein. Lane 1-uninduced strains containing pGEX4T-1 vector; Lane 2-induced strains containing pGEX4T-1; Lane-3-uninduced strains containing GmZF1::GST fusion; Lane 4-induced strains containing GmZF1::GST fusion; Lane 5-purified target protein as indicated by arrows. b: Probe design and composition. Nucleotide sequences of EP1S and mutated EP1S (E1, E2 and E3) probes. The nucleotide mutations in the EP1S core motif for each probe are boxed. c: The gel shift assay was performed in a solution containing 0.2 µg GmZF1::GST proteins or GST proteins and the probes ^32^P-labeled EP1S (E1) or mutant EP1S (E2 and E3), respectively. The GmZF1::EP1S complex and free probes are indicated by arrows.

Analysis of SDS-PAGE indicated that the GmZF1::GST fusion protein with 45.2 kD molecular weight was successfully expressed in *E. coli* strain (Fig. 2a), and the size of expressed protein is identical with the predicted molecular weight of target protein in the fusion vector vector pGEX-4 T-1. As a DNA-binding motif, a C2H2-type zinc-finger domain in the GmZF1 has been identified in many transcription factors. In our study, we observed that the transcription factors containing the zinc finger motif could specifically bind to EP1S core sequence (TGACAGTGTCA), which was originally identified as a *cis*-element within the *EPSPS* gene promoter in petunia [13], [23]. For identifying DNA-binding ability of GmZF1, a gel-shift assay was performed *in* *vitro* using the procedure as described in the method. Data showed that GmZF1 protein was expressed as fusion proteins with GST in *E. coli.* To better understand the molecular mechanism of specific-binding between the GmZF1 proteins and EP1S core sequences, a series of synthesis probes including wild type and mutants was prepared as the following descriptions. Briefly, the EP1S core sequence (wild-type, E1) and two probes (E2: bases substitution, E3: with four bases substitution) in the repeated sequences were prepared (Fig. 2b), and fusion protein was purified for gel shift assay. Assay showed that the GmZF1::GST fusion protein strongly bound to E1 and E2 probes, but weakly binding to E3 probe (Fig. 2c), no complex was found in reaction solution containing GST protein and probes, suggesting that GmZF1 protein could bind specifically to the EP1S core sequence *in* *vitro*, and two amino acid residues, G and C boxed in core sequence of TGACAGTGTCA probably play key role in recognizing DNA-binding sites in GmZF1 proteins.

### Subcellular localization of GmZF1

**Figure 3:**
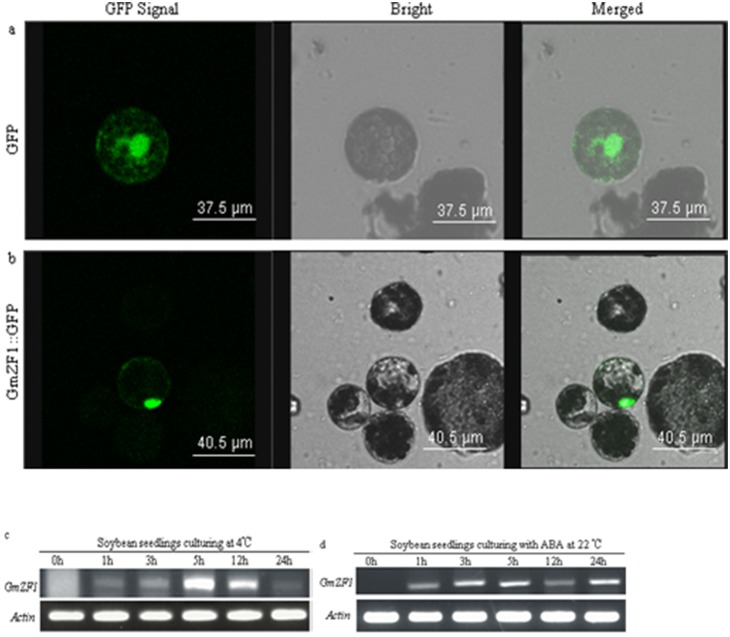
Subcellular localization of the GmZF1 proteins and mRNA accumulation of *GmZF1* gene in soybean seedlings respectively exposed to cold and ABA. a: Images expressing h16318-GFP in protoplast cells; b: images expressing the GmZF1::GFP fusion protein, fluorescent-field illumination was used to examine GFP signal (left); followed by bright-field illumination (middle) and confocal microscopy (right) for an merged image of bright and fluorescent illumination. c: the relative accumulation of *GmZF1* mRNA in soybean seedlings of 10-day-old exposed to 4°C at different time points; d: the accumulation of GmZF1 mRNA in soybean seedlings of 10-day-old exposed to ABA of 200 µM at different time points.

To identify the subcellular localization of GmZF1, we fused the full length of GmZF1 to GFP vector under control of the constitutive 35S promoter. Both the recombinant DNA constructs encoding a GmZF1::GFP fusion protein and a GFP protein were respectively introduced into *Arabidopsis* protoplast cells. Localization of the GmZF1::GFP fusion protein was visualized exclusively in the nucleus (Fig. 3b), whereas the control GFP (35S::GFP) was distributed throughout the protoplast cells (Fig. 3a), demonstrating that GmZF1 is a nuclear protein possibly functioning as a transcriptional activator.

### Expression of *GmZF1* in response to stress

To examine the transcription profile of *GmZF1* in soybean under cold stress, the expression of *GmZF1* in soybean was detected using semi-quantitative RT-PCR. The result showed that *GmZF1* was weakly expressed in young seedlings, and induced by low temperature and reached a maximum at 5 h after 4°C stress (Fig. 3c). Data also showed that *GmZF1* gene could be induced by exogenous ABA, and seems to have similar expression pattern under cold stress, and maintained at a high expression level when the seedlings were kept at ABA treatment (Fig. 3d), suggesting that *GmZF1* might be involved in plant responses to cold stress via an ABA-dependent pathway.

### Phenotype changes of plants exposed to cold stress

**Figure 4:**
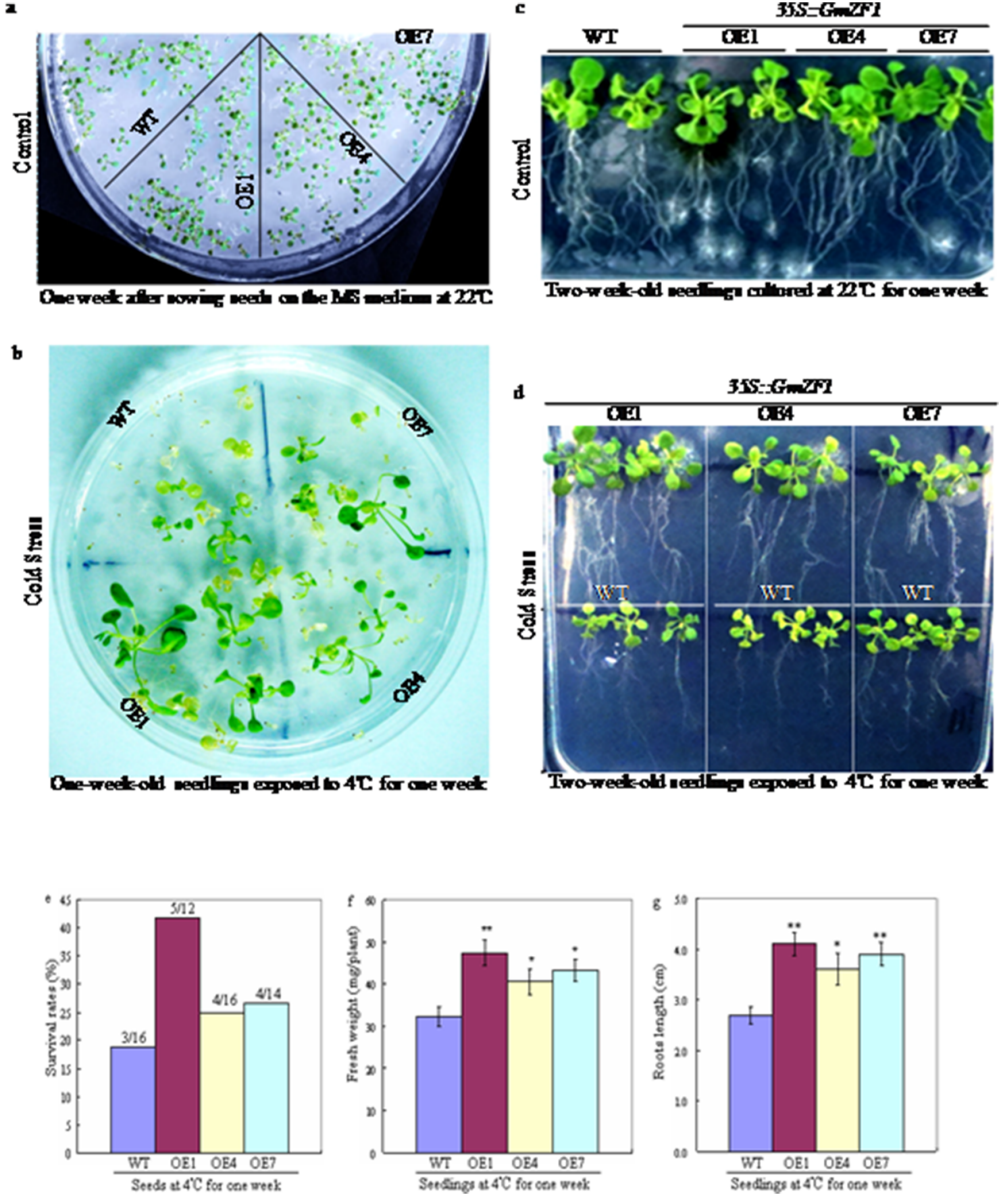
Morphological identification on wild type and transgenic *Arabidopsis* exposed to cold stress at 4°C on the MS medium. a: *Arabidopsis* seedlings of one-week-old after culturing at 22°C; c: *Arabidopsis* seedlings of two-week-old cultured at 22°C for one week; b and d: *Arabidopsis* seedlings of one-week-old cultured at 4°C for one week or two weeks, respectively; e: the survival rates of *Arabidopsis* seedlings exposed to 4°C for one week; f: the fresh weight per plant of two-week-old *Arabidopsis* after culturing at 4°C for one week; g: the roots length of two-week-old *Arabidopsis* seedlings after culturing at 4°C. WT-wild type; OE1, OE4 and OE7- transgenic lines.

To observe the difference in germination and survival rates between the wild type and the transgenic *Arabidopsis*, two independent culture experiments was performed at 22°C and at 4°C, respectively (Fig. 4a, b) Fig. 4a showed that both wild type and transgenic lines almost exhibited identical germination rates under favorable condition, but the survival rate of transgenic line OE1 was significantly higher than that of wild types under cold stress when wild type and transgenic lines of one-week-old seedlings were exposed to 4°C for one week (Fig. 4e). For observing the phenotypic changes of wild type and transgenic *Arabidopsis* under cold stress, the seedlings of two-week-old were transferred onto the MS medium, and cultured at 22°C (Fig. 4c) and 4°C (Fig. 4d) for one week, respectively (note: white bacterial plaques appeared in the mediums because of possible contaminant from culture processes). Data showed that transgenic lines showed a better phenotypic characteristic in withstanding cold stress compared to wild types (Fig. 4d), because both fresh weight and root lengths from the transgenic lines were significantly increased comparing to the wild types (Fig. 4f, g). These data suggested that *GmZF1* in the transgenic *Arabidopsis* could enhance the tolerance plants to the cold stress. *GmZF1* and *GsZFP1* [22] seems to play similar roles in acclimating the tolerance of plants to cold stress, although these two genes have differences in the structure, especially in the conserved domains of C2H2 zinc finger gene, suggesting that different zinc finger genes in the same super family could demonstrate an uniformity in gene functions even though they have the difference in the genetic characteristics, and this seems to imply that the diversity genetic of genes is an essential factor in regulating the expression of target genes related to abiotic stresses. Therefore, to better elucidate the molecular regulation mechanism of *GmZF1* from cultivar and *GsZFP1* from wild soybean in responding to cold stress, further investigations is necessary.

### Over-expression of *GmZF1* led to physiological changes in the transgenic lines under cold stress

**Figure 5:**
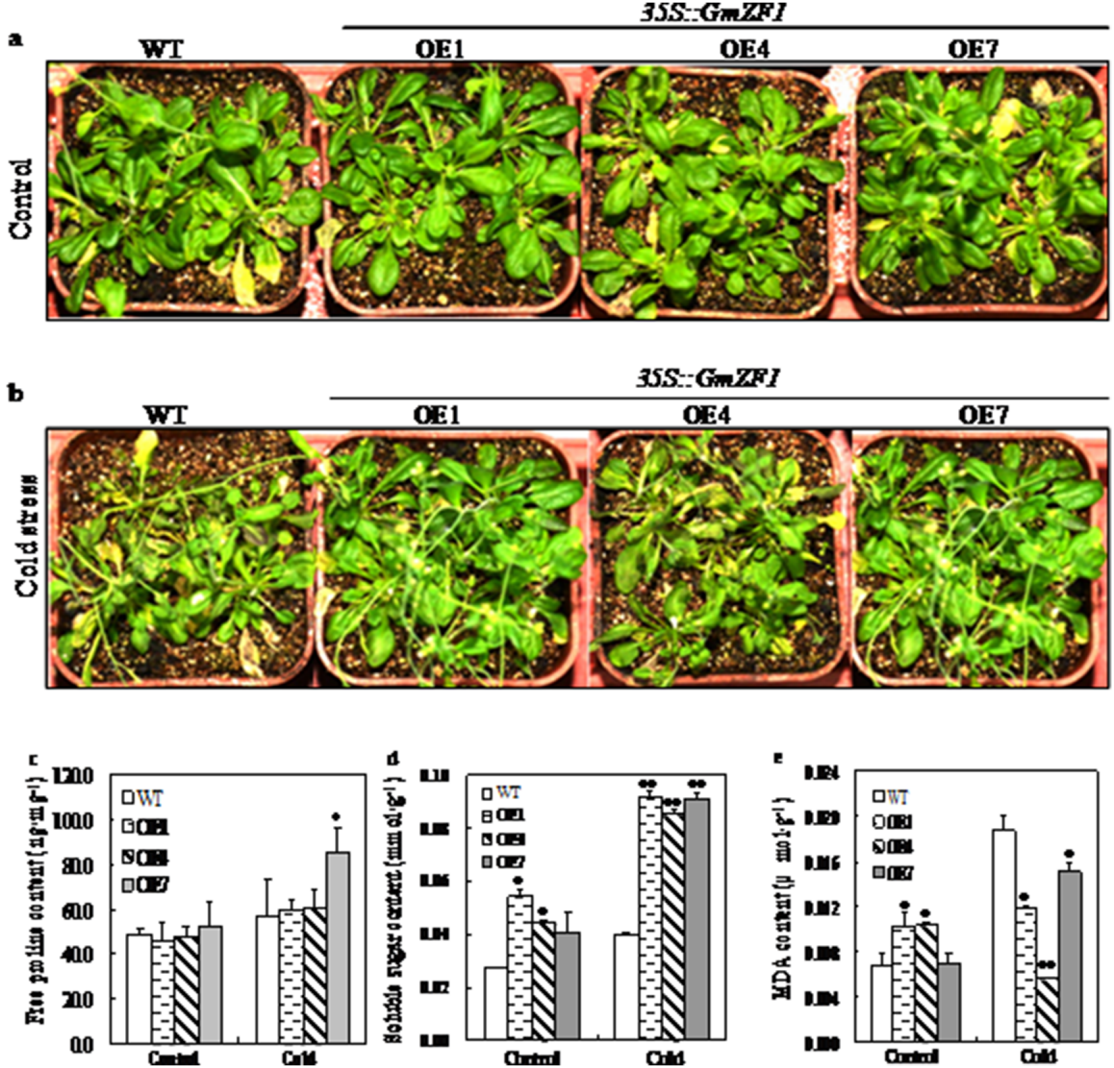
Phenotypic and physiological changes in wild type and transgenic *Arabidopsis* lines under cold stress. a: Phenotypes of wild type and transgenic lines of 5-week-old seedlings before cold stress (Control). b: Phenotypes of wild type and transgenic lines of 5-week-old seedlings exposed to 4°C for one week (Cold stress). c, d and e respectively represent the content changes of free proline, soluble sugar and MDA in the leaves of wild type and in transgenic lines before (Control) and after cold stress (Cold). WT-wild type; OE1, OE4 and OE7-transgenic lines**;** single * and duble ** respectively means significant difference at 0.05 and 0.01 levels.

To further characterize the function of *GmZF1*, we generated *Arabidopsis* transgenic plants carrying *35S::GmZF1*, in which *GmZF1* was driven by the CaMV35S promoter. Total 22 transformed *Arabidopsis* plants with kanamycin-resistant were obtained, and three independent homozygous transgenic lines (OE1, OE4 and OE7) carrying *GmZF1* were continuously selected by the PCR analysis until T3 transgenic *Arabidopsis* lines. For cold stress, 5-week-old wild-type and transgenic lines were transferred to the incubator at 4°C for two days, and morphological observation showed that the transgenic lines were not significantly inhibited in growth comparing to wild type in spite of being a short term cold stress, and the growth of the transgenic lines carrying *GmZF1* gene were slightly damaged comparing to wild type (Fig. 5a, b). However, three transgenic lines have remarkable physiological changes in accumulation of the proline, soluble sugar and MDA in the leaves comparing to the wild type after cold stress. Cold stress enhanced accumulation of proline and soluble sugar in the wild type and transgenic lines, but the increase times in the contents of proline in the transgenic lines (OE1, 1.3-fold; OE4, 1.3-fold and OE7, 1.6-fold) were significantly higher than that in the wild type (1.1-fold) (Fig. 5c). Similarly, the increase times in the content of soluble sugar in the leaves from OE1, OE4 and OE7 (1.7-fold, 1.9-fold and 2.2-fold) were also higher than that in the wild type (1.4-fold) after cold stress for two days (Fig. 5d). In contrast, the contents of MDA in the transgenic lines (OE1, 0.012 µmol·g^−1^; OE4, 0.006 µmol·g^−1^ and OE7, 0.015 µmol·g^−1^) were decreased comparing to the wild type (0.019 µmol·g^−1^) after cold stress (Fig. 5e), demonstrating that over-expression of *GmZF1* reduced oxidative damage of membrane lipid peroxidation, and further maintained osmotic balance in the plant cells.

**Figure 6:**
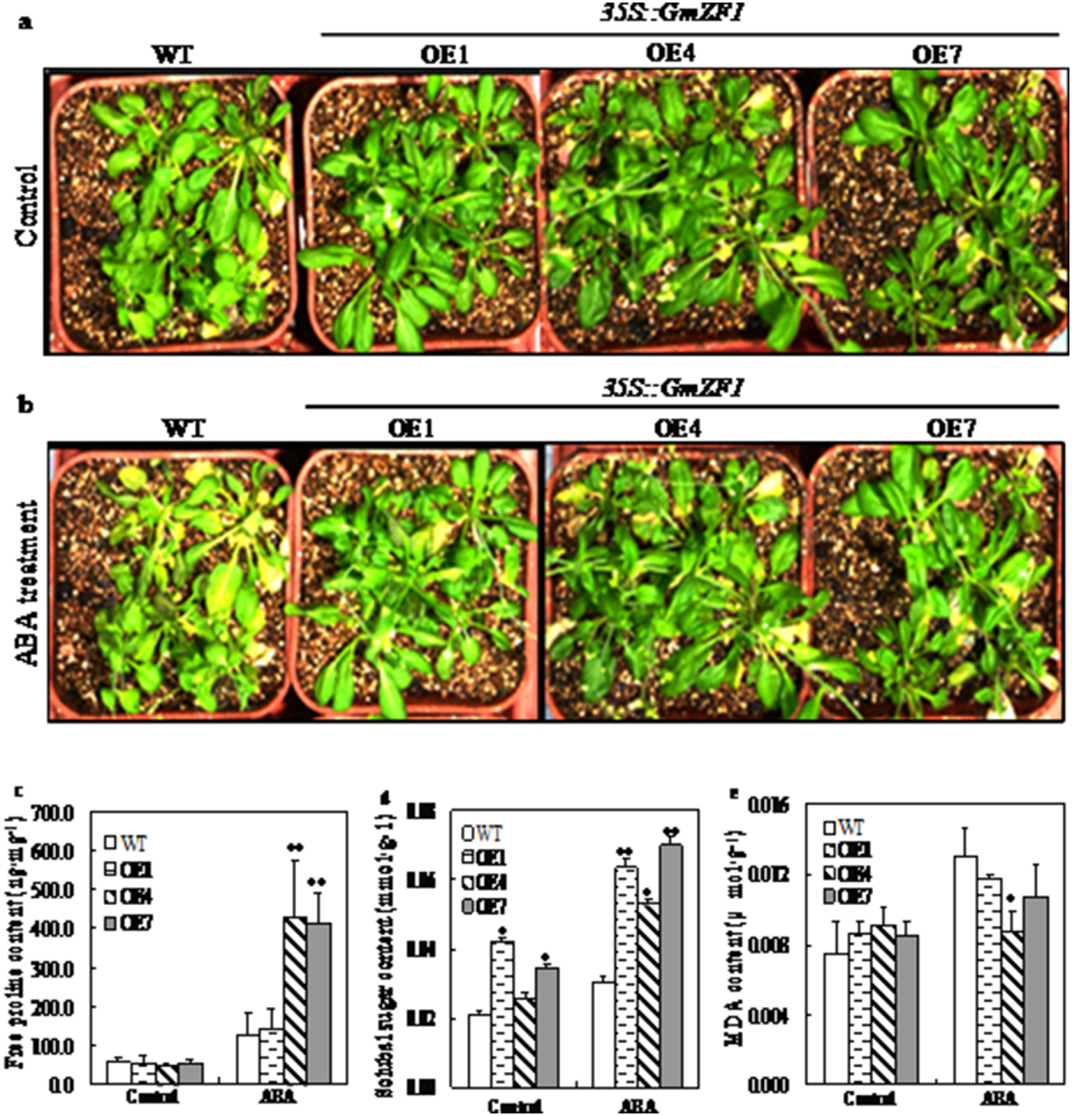
Phenotypic and physiological changes in wild type and transgenic *Arabidopsis* lines exposed to ABA. a: Phenotypes of wild type and transgenic lines of 5-week-old seedlings before ABA treatment. b: Phenotypes of wild type and transgenic lines of 5-week-old seedlings exposed to 200 µM ABA for one week. c, d and e respectively represent the content changes of free proline, soluble sugar and MDA in the leaves of wild type and transgenic lines before (Control) and after ABA treatment (ABA). WT-wild type; OE1, OE4 and OE7-transgenic lines**;** single * and duble ** respectively means significant difference at 0.05 and at 0.01 levels.

To observe the physiological effects of exogenous ABA supply on the transgenic *Arabidopsis* lines, the leaves from the wild type and the transgenic *Arabidopsis* lines exposed to ABA solution was sampled, and used for the determination in the content changes of praline, soluble sugar and MDA. Data showed that the contents of proline in the wild type were almost identical with that in the transgenic lines before ABA treatment. However, the content of proline in the transgenic lines (OE1, 142.4 ng·mg^−1^; OE4, 427.2 ng·mg^−1^ and OE7, 412.9 ng·mg^−1^) were significantly higher than that in the wild type (125.7 ng·mg^–1^) after application of ABA (Fig. 6c). In addition, ABA treatment also enhanced the levels of soluble sugar in the wild type and the transgenic lines, but the increase times in the content of soluble sugar in the transgenic lines (OE1, 1.5-fold; OE4, 2.1-fold and OE7, 2.0-fold) were higher than that in the wild type (1.4-fold) after two days by ABA treatment for (Fig. 6d). The contents of MDA in the transgenic lines (OE1, 0.012 µmol·g^−1^; OE4, 0.009 µmol·g^−1^ and OE7, 0.011 µmol·g^−1^) were reduced comparing to the wild type (0.013 µmol·g^−1^) after ABA treatment, whereas the contents of MDA in the transgenic lines (OE1, 0.009 µmol·g^−1^; OE4, 0.009 µmol·g^−1^ and OE7, 0.008 µmol·g^−1^) were slightly higher than that in the wild type (0.007 µmol·g^−1^) before ABA treatment (Fig. 6e).

**Figure 7:**
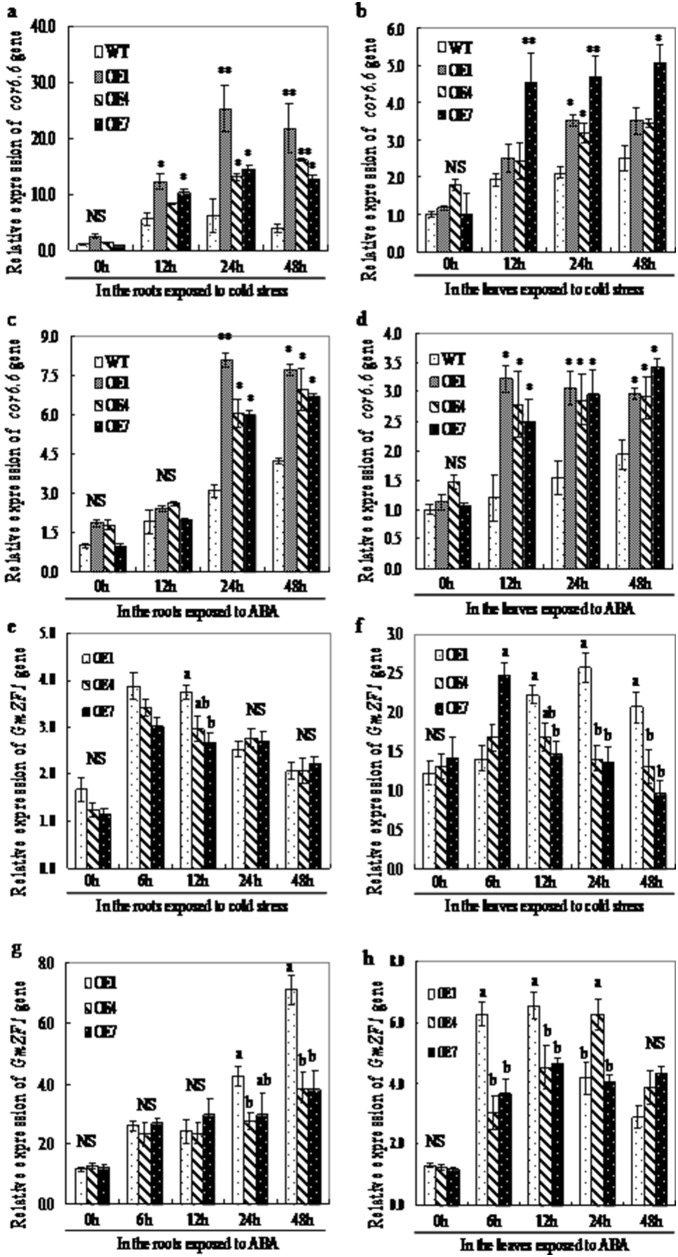
Responsive expression of *cor6.6* gene and relative accumulation of *GmZF1* mRNA in wild type and transgenic lines both under cold stress and ABA supply. a and b respectively represent the relative expressions of *cor6.6* gene in the roots and leaves of plants exposed to cold stress; c and d respectively represent the relative expressions of *cor6.6* gene in the roots and leaves of plants exposed to ABA, and the expression level of *cor6.6* gene in unstressed wild type was set at 1.0 in the transgenic *Arabidopsis* lines. e: the relative expressions of *GmZF1* gene in the roots; f: the relative expressions of *GmZF1* gene in the leaves of transgenic plants exposed to cold stress; g and h respectively represent the relative expressions of *GmZF1* gene in the roots and leaves of transgenic plants exposed to ABA. Error bars indicate the standard deviation; NS means no significant differences; lowercase letters labeled on the columns mean statistical difference at 0.05 level ce at 0.05 level; single * and duble ** respectively means significant difference at 0.05 and at 0.01 levels; WT-wild type; OE1, OE4 and OE7-transgenic lines.

The induction of numerous stress-responsive genes is a hallmark of stress acclimation in plants. To elucidate the molecular mechanism of *GmZF1* in responding to cold, both wild type and transgenic seedlings of *Arabidopsis* were incubated at 4°C or by ABA for 48 hours to examine the expression of cold-responsive genes *cor6.6* at different time points by real-time PCR analysis. As shown in Fig. 7, both wild type and transgenic lines (OE1, OE4 and OE7) exhibited a similar levels in the expression of *cor6.6* before cold treatment, and the OE1 line began to appear significant up-regulations in *cor6.6* expressions in the roots after 12 hours cold treatment, while the OE7 line significantly increased the expression of *cor6.6* gene in the leaves after 12 h cold stress (Fig. 7a, b) When the seedlings of *Arabidopsis* were exposed to exogenous ABA, the expressions of *cor6.6* gene in the roots of the OE1 and OE7 lines were significantly induced after 24 hours (Fig. 7c), and the all transgenic lines (OE1, OE4 and OE7) exhibited remarkable increase in the expression of *cor6.6* gene in the leaves after 12 hours (Fig. 7d). Although both the wild type and transgenic lines at some time points did not exhibit significant differences in the expression of *cor6.6*, but the transgenic lines, OE1, OE4 and OE7 still showed higher transcript levels of *cor6.6* gene than the wild type under cold stress or exogenous ABA supply (Fig. 7b, c and d), suggesting that *GmZF1* may play an important role in activating cold-resistance genes in the transgenic *Arabidopsis* responding to cold stress through an signal pathway depending ABA.

Fig. 7e, f showed that the expressions of *GmZF1* gene in the roots and in the leaves of transgenic lines significantly increased after exposing the plants to cold stress for 6 hours, and this result seems to be identical with that in soybean seedlings exposed to cold stress or ABA (Fig. 3c, d). Fig. 7e showed that the expression of *GmZF1* gene in the roots of OE4 and OE7 was almost identical, but the OE7 line exhibited obvious increase in the expression of *GmZF1* gene in the leaves after 6 hours cold stress relative to other transgenic lines, and then maintained at relative stable levels at other time points (Fig. 7f). In addition, accumulation pattern of *GmZF1* gene in the transgenic *Arabidopsis* lines treated by exogenous ABA also showed that *GmZF1* gene in the leaves of the transgenic *Arabidopsis* lines was strongly induced by ABA, and the accumulation levels *GmZF1* gene in the roots of OE1 line were increased significantlyafter exposing to ABA for 24 hours (Fig. 7g) hours, and the accumulation of *GmZF1* mRNA in the leaves of OE the transgenic lines exhibited strong induction by ABA after 6 hours, and only OE7 line has almost identical induction accumulation patterns of *GmZF1* mRNA by ABA, and the induction accumulation seems to be inhibited after 48 hours (Fig. 7h). However, the accumulation of *GmZF1* mRNA in the OE7 transgenic line was little after exposing to ABA supply compared to the other transgenic lines, indicating that different transgenic lines exhibited different accumulation patterns in expressions of *GmZF1* gene in the plant organs, and seems to imply that accumulation patterns of *GmZF1* mRNA in the transgenic plants probably are related to the positions and copy numbers of *GmZF1* gene inserting in the genome of *Arabidopsis*.

## Discussion

The C2H2 zinc-finger transcription factors were usually thought to be involved in plant development and have various adaptive responses to the environment stress [10], [24]. Although the roles of some C2H2 zinc-finger transcription factors have been identified to be related to stress and developmental processes, the functions of C2H2 ZFPs from soybean involved in stress response are largely unknown [22].

In this study, as a novel C2H2 zinc finger protein gene, *GmZF1* from soybean was characterized. Sequence analysis revealed that the GmZF1 had high identity with other C2H2-type ZFPs, and shared two zinc finger motifs containing a conserved plant-specific QALGGH amino acid sequence which was proved to be critical for DNA-binding activity [11]. Based on the present data, we predicted that the binding activity of GmZF1 to EP1S core elements was probably mediated by the QALGGH sequence. Additionally, the C-terminus of *GmZF1* gene contains typical Leu-rich L-box and DLN-box which play roles in protein interactions or in maintaining the folded structure [19]. The DLN-box was thought to function in transcriptional repression. As reported previously, the DLN-boxes in the zinc finger proteins ZPT2–3 from petunia [16] and STZ/ZAT10 from *Arabidopsis* exhibited repression roles in transcription activities [18]. However, some zinc finger proteins containing the DLN-box were involved in transcriptional activation, such as ThZF1 from *Thellungiella halophila* [25] and CaZF from chickpea [26]. Subcellular localization analysis revealed that GmZF1 localized at nuclei (Fig. 3), implying that GmZF1, like other ZFPs from plant TFIIIA-type, functions as a transcription factor in plant cells and may play an important role in signaling pathway in soybean under abiotic stress.

As a key regulator, ABA plays an important role in signaling pathways under stresses, such as drought, low temperature and osmotic stress, and induces the expressions of a number of genes that respond to abiotic stress [30], [31]. For example, the expression of *AZF2* gene from *Arabidopsis* was strongly induced following ABA treatment under drought and salt stress [1], [12], [18]. However, it was reported that some C2H2-type zinc finger genes are induced by dehydration and cold stress, but do not respond to exogenous ABA [2], [32], [33], suggesting that two kinds of signaling transduction pathways, ABA-independent pathway and ABA-dependent pathway play different regulation roles in responding to abiotic stresses, repectively, and that the initial stress signal was converted into cellular responses [34]. In this study, our data proved that *GmZF1* was involved in plant responses to cold stress through an ABA-dependent signal transduction pathway since the expression of *GmZF1* in soybean seedlings was clearly induced by ABA (Fig. 4). In addition, accumulation pattern of *GmZF1* gene in the transgenic *Arabidopsis* lines treated by exogenous ABA also showed that over-expression of *GmZF1* gene in *Arabidopsis* was strongly induced by ABA.

Some of the C2H2-type ZFPs from different plant species are confirmed to play regulatory roles in stress responses, such as AZF2 and STZ in Arabidopsis [12], [17], [27], SCOF-1 in soybean [21], GsZFP1 in wild soybean [22], StZFP1 in potato [9], DST in rice [28] and TaCHP in wheat [29]. Expression analysis revealed that GmZF1 was clearly induced by cold stress (Fig. 4), suggesting that *GmZF1* might be involved in plant responses to cold stress in soybean. Transgenic *Arabidopsis* plants over-expressing *GmZF1* were evaluated for the involvement of *GmZF1* gene in cold tolerance of plants. Although no morphological differences between the transgenic lines and the wild type was observed, the remarkable differences in accumulation of free proline, soluble sugar and MDA were confirmed after cold and ABA treatment (Fig. 5c–e and Fig. 6c–e). Accumulation of proline by stress-induced has been observed in many plant species, and functions as an osmo-protectant coping with stress [32], [35], [36]. For example, the transgenic tobacco plants over-expressing *GmDREB3* could accumulate much free proline than the wild-type plants after drought stress treatment for 16 d [37]. In plants, soluble sugar has been shown to fulfill a dual role as both metabolites and as signaling molecules [38], [39] that may play important roles in the mechanisms of plant responding to the stress [40], [41]. In our study, the accumulations of proline and soluble sugar in the wild type and transgenic lines were improved, and increase times in the contents of proline and soluble sugar in the transgenic lines were higher than that in wild type during cold and ABA treatment (Fig. 5 and Fig. 6), proving that the accumulation of soluble sugars has been occurred in many plant species during cold acclimation [42]. Based on our investigations, both wild type and transgenic *Arabidopsis* lines basically have no significant differences in germination rates. However, the survival rates from these two phenotypes was remarkable when these two seedlings were exposed to cold stress, the survival rates of transgenic *Arabidopsis* lines cultured at 4°C significantly increased after one week comparing to the wild types (Fig. 4 a,b), indicating that *GmZF1* gene could play regulation role during the plant growth and development. Our study showed that over-expression of *GmZF1* gene significantly enhanced the transcription levels of *cor6.6* gene responding to cold stress and ABA supply, and increased the accumulation of proline, soluble sugar in the transgenic *Arabidopsis*, and reduced the content of MDA in the transgenic lines comparing to the wild type, and significantly improved the tolerance of the transgenic lines exposed to cold stress and exogenous ABA (Fig. 5e; Fig. 6e). In conclusion, over-expression of *GmZF1* resulted in an enhancement in cold tolerance of the transgenic *Arabidopsis* by activating transcription of *cor6.6* and/or cold resistance-genes (Fig. 7) as well as accumulation changes of proline, soluble sugar and MDA [43]–[45]. Bioinformatics analysis fund that the promoter with 1955 bp of *cor6.6* gene contains 7×GACA and 6×GTCA repeat in core element TTGACAGTGTCAC, respectively (NCBI Reference Sequence in *Arbidopsis thaliana* chromosome 5: NC_003076.8-AT5G15950/NM_121600.3/NP_197099.1), and these two core elements possibly play key role in recognizing DNA-binding sites in target proteins, because the mutation of G and C in the two boxed core elements could change the ability of GmZf1 protein binding to DNA, especially the mutation of G and C in the GCTA box (Fig. 3c). Therefore, *GmZF1* could play regulation role by activating the transcription of *cor6.6* cold-regulated gene in *Arabidopsis,* and led to an enhancement in the tolerance of transgenic plants to cold stress.

## Materials and Methods

### Plant materials and growth conditions

Soybean seedlings (*Glycine max*) were grown in a growth chamber at 24°C with 60% relative humidity under 16 h light and 8 h darkness. *Arabidopsis* plants (genotype Colombia) were grown in a controlled environmental chamber at 22°C and 70% humidity with a 14 h light/10 h darkness cycle under normal light intensity (150 Em^−2^·s^−1^). T_1_ seeds were sterilized and planted on the MS medium containing kanamycin of 50 µg·mL^−1^ for the selection of transgenic plants. After continuously screening on the MS medium containing kanamycin of 50 µg·mL^−1^ for two times and the generated T3 seedlings of transgenic *Arabidopsis* lines were transplanted to pots or MS medium for further investigations.

### Isolation and sequence analysis of *GmZF1*

The zinc finger gene *GmZF1* was cloned from soybean based on the expressed sequence tag (EST) database of soybean. Total RNA was extracted from soybean seedlings using RNA Prep. Pure plant kit (Tiangen Biological Company, Beijing), and cDNA synthesis was performed by a reverse transcription kit (TaKaRa Dalian BioCompany). The entire *GmZF1* cDNA of was obtained by PCR using the specific primers (F: 5′-AGAGGAAACTAGCTAGGGCACTTC-3′ and R: 5′-CCCGAGAACTAAGAAGTTTCGTATT-3′). The deduced amino acid sequences of soybean GmZF1 zinc finger protein were matched by a protein blast procedure (http://www.ncbi.nlm.nih.gov/BLAST/). BioEdit version 7.0 software was used to multiple sequence alignments. Phylogenetic analysis was carried out by MEGA version 4.0 with adopting position correction distance using a bootstrap replicate number of 1000.

### Induction expression of *GmZF1* fusion protein and electrophoretic mobility shift assay

An entire 516-bp *GmZF1* fragment containing the DNA binding domain was amplified by PCR using a pair of primers F and R (F: 5′-ACAACTCGAGATGAAGAGAGGCAGAGAA-3′ and R: 5′-AGACGAATTCAATGAAACAATTGAGCAC-3′), and was subcloned onto the pGEX4 T-1 vector by inserting at two specific sites, *EcoR*I and *Xho*I (Amersham Biosciences), and the recombinant pGEX-4 T-1 plasmids were identified by sequencing, and transferred into *Escherichia coli* BL21 cells (Amersham Biosciences). The induction of target fusion proteins was performed by adding 0.8 mM IPTG (Isopropyl-β-d-thiogalactopyranoside) into the culture of *E. coli* strains, and the cultures carrying GmZF1::GST fusion proteins was incubated for 5 hours at 37°C with 250 rpm The bacterial cells were shattered by ultrasonication in phosphate-buffered saline (PBS) and centrifuged at 11000 *g* for 10 min to remove insoluble cell debris, and then the supernates were collected, and applied on the gel containing 12% ployacrylamide for electrophoresis detection of fusion protein by the method of SDS-PAGE described in the book [46]. The GST::GmZF1 fusion protein was purified using a glutathione-Sepharose 4B column (Amersham Biosciences) according to the manufacturer’s instructions. The 52 bp DNA fragment containing four copies of wild-type or mutant EP1S core sequences were labeled by 25 µCi·µL^−1^ of γ-^32^P-dATP (Amersham Biosciences). The DNA-binding reaction was performed in a 20 µL binding buffer containing ^32^P-labelled probe, glycerine and purified fusion protein [24], and were subjected to electrophoresis with the gel containing 0.53 Tris-borate-EDTA, 6% polyacrylamide, and the gels were dried and visualized by autoradiography.

### Subcellular localization of *GmZF1* gene

In brief, an entire *GmZF1* cDNA fragment was amplified by PCR procedure with a pair of specific primers (F: 5′-AACACTGCAGATGAAGAGAGGCAGAGA-3′), and R: 5′-CGGGATCCAATGAAACAATTGAGCAC-3′.). Based on the specific sites in the MCS of GFP vector, two specific sites, a *Pst*I and *BamH*I were respectively inserted into the 5′ ends of the primers for subcloning requirement. The digested PCR fragment was inserted into the multiple cloning sites (MCS) in the GFP vector carrying a GFP protein driven by a 35 S promoter, and this construct carrying 35 S::GFP or 35 S::GmZF1::GFP were introduced into *Arabidopsis* protoplast cells, which was prepared by young seedlings of *Arabidopsis,* and the transformation procedures were performed by the method as described previously [47], [48]. The images were captured using a Nikon Eclipse TE2000-U microscope (Nikon).

### Transformation of *Arabidopsis*

A pair of specific primers, the *GmZF1* cDNA fragment was generated by PCR procedure using the specific primers (F: 5′-AACACCATGGCCATGAAGAGAGGCAGAGA-3′, and R: 5′-AGACACTAGTAATGAAACAATTGAGCAC-3′. The generated PCR product directly was inserted onto the pCAMBIA1304 vector using a pair of specific restriction sites (*Nco*I and *Spe*I), and an over-expression vector p35 S::GmZF1 was constructed, and introduced into *Agrobacterium tumefaciens* strain C58C1. The flowering *Arabidopsis* seedlings were used for genetic transformation at 25°C by the method of floral-dip. T3 transgenic *Arabidopsis* plants were continuously cultured in the medium containing kanamycin of 45 mg·L^−1^ and three positive transgenic *Arabidopsis* were identified and the T3 seeds of transgenic *Arabidopsis* lines and wild type *Arabidopsis* were sowed on MS medium based on the requirements of experiment, and the resulting seedlings were accordingly transferred into the pots and MS medium for cold stress and ABA treatment.

### Stress treatments

Cold stress was performed by incubating soybean seedlings of 10-day-old at 4°C for 24 hours and *Arabidopsis* seedlings of two-week-old seedlings were incubated at 4°C for 48 hours. ABA treatment was respectively carried out by completely spraying 200 µmol·L^−1^ ABA onto the 10-day-old soybean seedlings and two-week-old *Arabidopsis* seedlings Soybean leaves from the same position were respectively sampled at 0, 1, 3, 5, 12 and 24 hours, and *Arabidopsis* leaves were collected respectively at 0, 6, 12, 24 and 48 hours. Equal amounts of seeds from wild type and transgenic lines were sowed on MS medium at 22°C (Control), and the generated seedlings of one-week-old *Arabidopsis* were cultured at 22°C and at 4°C (Cold stress), respectively, for one week in two independent growth chambers, and used for investigation in survival rates. For evaluating the changes of Arabidopsis biomass and root development under cold stress, two-week-old seedlings of *Arabidopsis* from control culture were transplanted on the MS medium, and cultured respectively at 22°C (Control) and at 4°C (Cold stress) for one week in two independent growth chambers, and biological fresh weights per plant and root lengths from two treatments were statistically investigated based on the three replicates.

### Gene expression analysis

Based on the equal amounts of leaves RNA from each time point, the expression pattern of *GmZF1* gene in soybean was characterized using semi-quantitative RT-PCR by GmF and GmR primers, and accumulation analysis of *GmZF1* mRNA in the transgenic *Arabidopsis* lines was performed using the specific primers (GmF: 5′-ATGAAGAGAGGCAGAGAA-3′ and GmR: 5′-AATGAAACAATTGAGCAC-3′) by quantitative real-time PCR (qPCR) For understanding the expression profile of GmZF1 gene and cold-responsive marker gene *cor6.6* in the transgenic *Arabidopsis* under low temperature stress and exogenous ABA supply, the wild type Arabidopsis and transgenic *Arabidopsis* lines carrying GmZF1 gene were incubated at 4°C for 48 hours in darkness. All samples including the roots and leaves were collected with three biological replicates at the designated time intervals after cold and ABA treatment, and were quickly frozen in liquid nitrogen and stored at 80°C for total RNA isolation. As a reference gene in *Arabidopsis*, actin was used as an internal maker, and amplified by the specific primers [(F 5′-AAGTATCCTATTGAGCATGGTGTTG-3′; R 5′- CTGGCGTACAAGGAGAGA-3′), (accession number: AEE76148)], and the *cor6.6* gene was amplified by the primers (F 5′- ATGTCAGAGACCAACAAGAATG-3′; R5′- CTTGTTCAGGCCGGTCTTG-3′) (accession number: CAA38894) with qPCR, which was performed using a SYBR premix Ex Taq kit (TaKaRa) according to the manufacturer’s instructions on a 7900HT Real-time PCR system.

### Accumulation analysis of free proline, soluble sugar and malondialdehyde (MDA)

0.5 g fresh leaves from each treatment of the wild-type and the transgenic *Arabidopsis* lines were respectively collected at different time point, and respectively were treated according to requirements of experiment and used for measurements of free praline, soluble sugar and MDA. The contents of free proline were measured by the previous procedure [40], [49], [50]. Measurement of MDA content in *Arabidopsis* leaves was determined as described previously [51], [52], and the content of soluble sugar was measured with the method as described by [53].
